# Distributed Consensus Estimation for Networked Multi-Sensor Systems under Hybrid Attacks and Missing Measurements

**DOI:** 10.3390/s24134071

**Published:** 2024-06-22

**Authors:** Zhijian Cheng, Lan Yang, Qunyao Yuan, Yinren Long, Hongru Ren

**Affiliations:** School of Automation, Guangdong-Hong Kong Joint Laboratory for Intelligent Decision and Cooperative Control, and Guangdong Provincial Key Laboratory for Intelligent Decision and Cooperative Control, Guangdong University of Technology, Guangzhou 510006, China; chengzhijian2019@163.com (Z.C.); yanglan202304@163.com (L.Y.); yuanqunyao2000@163.com (Q.Y.); longyinren2022@163.com (Y.L.)

**Keywords:** networked multi-sensor systems, distributed consensus estimation, hybrid attacks, missing measurements

## Abstract

Cyber-security research on networked multi-sensor systems is crucial due to the vulnerability to various types of cyberattacks. For the development of effective defense measures, attention is required to gain insight into the complex characteristics and behaviors of cyber attacks from the attacker’s perspective. This paper aims to tackle the problem of distributed consensus estimation for networked multi-sensor systems subject to hybrid attacks and missing measurements. To account for both random denial of service (DoS) attacks and false data injection (FDI) attacks, a hybrid attack model on the estimator-to-estimator communication channel is presented. The characteristics of missing measurements are defined by random variables that satisfy the Bernoulli distribution. Then a modified consensus-based distributed estimator, integrated with the characteristics of hybrid attacks and missing measurements, is presented. For reducing the computational complexity of the optimal distributed estimation method, a scalable suboptimal distributed consensus estimator is designed. Sufficient conditions are further provided for guaranteeing the stability of the proposed suboptimal distributed estimator. Finally, a simulation experiment on aircraft tracking is executed to validate the effectiveness and feasibility of the proposed algorithm.

## 1. Introduction

With the advancement of communication technologies, networked multi-sensor systems have garnered significant interest in recent decades [1,2]. Networked multi-sensor systems contain components connected via a shared network, thus reducing unnecessary wired connections, lowering installation costs, and increasing system scalability [3,4,5]. It is because of such benefits that networked multi-sensor systems are extensively applied in smart grids, autonomous driving, robotics, and satellite navigation [6,7,8]. However, due to the data transmitted over open and shared communication links, networked multi-sensor systems are vulnerable to malicious cyber attacks, which can pose a huge threat to life and property security [9]. As a result, it is of utmost importance to enhance the security of networked multi-sensor systems to ensure their normal operation. This issue has attracted widespread attention in recent years [10,11,12].

There are two main categories into which typical attack models in networked multi-sensor systems fall: denial of service (DoS) attacks and deception attacks [13]. As all individuals know, false data injection (FDI) attacks, which are regarded as a typical deception attack, seek to manipulate the transmitted data by injecting some faked data [14]. DoS attacks attempt to prevent legitimate users from accessing the server by sending a great deal of false information, thereby blocking the communication channel [15]. Obviously, both types of cyber attacks can have profound negative impacts on networked multi-sensor systems. This problem has also aroused considerable interest among researchers, especially regarding the estimation and control issues under FDI and DoS attacks. For instance, based on prior research in [14], the author proposed a power system state estimation algorithm under imperfect FDI attacks. The FDI attack model in [14] aimed at compromising defenseless sensors to corrupt measurement information, focusing on the stealthiness of the attack strategy. By utilizing the event-triggered mechanism, a modified secure remote estimator under DoS attacks was designed for cyber-physical systems [16], in which DoS attacks occurring on the sensor-estimator communication channel considered noise and interference. It is common to find only a single type of attack considered in estimation for sensor networks. However, in practical systems, to increase the possibility of success of attacks, adversaries often alternately launch different types of attacks with a certain probability [13]. Such hybrid attacks not only have a greater negative impact on estimation and control algorithms, but also pose challenges to existing attack detection mechanisms [17]. Therefore, this has aroused the interest of researchers to address the estimation and control issues of networked multi-sensor systems under hybrid attacks.

As mentioned before, previous work focused more on centralised multi-sensor systems or single sensor systems, rather than on distributed systems. With the all-round development of computation and communication capabilities in sensor networks, distributed estimation is widely applied in networked multi-sensor systems due to its high robustness, scalability and flexibility [9,18]. However, since information sharing and data transmission are constrained by the inherent coupling relationship between different nodes, distributed sensor networks are more vulnerable to various cyber attacks [19,20]. As a result, it is a critically important yet complicated topic to investigate the security issues of distributed multi-sensor systems. In most existing research, some results such as [21,22,23] primarily addressed the distributed estimation problem under malicious cyber attacks on communication links connecting sensors, and only considering a single type of attack. To our knowledge, however, few research have addressed the distributed consensus estimation problem under hybrid attacks that occur between estimators, where data transmitted over wireless networks between nodes may be tampered with by attackers.

Note that distributed consensus estimation is formed by integrating multi-agent consensus theory into the standard Kalman filter, so it also faces the challenge of missing measurement issues in traditional state estimation. This challenge has spawned a large amount of related research [24,25,26]. For instance, a novel locally optimal distributed consensus estimator was presented in [25] for stochastic systems with missing measurements, where the missing measurement phenomena are represented by a set of random variables with Bernoulli distribution. However, reviewing the literature on distributed estimation from the past few years, it is rare to find that issues regarding cyber attacks and network communication such as missing measurements are taken into account simultaneously. This is mainly because the superimposed effect of missing measurements and cyber attacks will accelerate the degradation of distributed estimation performance, eventually leading to system instability.

Drawing from the aforementioned discussions, this paper focuses on distributed consensus estimation issues for networked multi-sensor systems subject to the dual impact of hybrid attacks and missing measurements. The following three points highlight the difficulties encountered in this paper: (1) how to construct a hybrid attack model targeting the estimator–estimator communication channel to account for the joint impact of FDI and DoS attacks? (2) how can the optimal filter gain matrix be determined under the influence of multi-random variables? (3) how to construct suitable sufficient conditions to ensure the convergence of the estimation error under the dual impact of hybrid attacks and missing measurements?

In light of these difficulties, the following is a summary of the main contributions in this paper:

1. Governed by multi-random variables with Bernoulli distribution, a unified hybrid attack model considering the joint impact of random FDI and DoS attacks is proposed. Different from cyber attacks on the sensor-to-estimator communication channel in [13,18], the proposed hybrid attack disrupts the data transmission between neighboring estimators in the distributed consensus estimation.
2. This paper is the first attempt to provide a modified distributed consensus estimation algorithm for networked multi-sensor systems subject to hybrid attacks and missing measurements. A suboptimal distributed estimation algorithm, simplified by an approximation method, is devised to circumvent the computation of the cross-covariance matrix, thereby reducing the computational complexity.
3. A co-design scheme of consensus gain coefficients, hybrid attack parameters, missing measurement probabilities and model parameters based on Lyapunov stability analysis is proposed. It is theoretically proved that the stability of the proposed distributed consensus estimator can be guaranteed by constructing a sufficient condition.

The following is how the rest of the paper is structured. System models under missing measurements, hybrid attack models, and problem descriptions on the distributed consensus estimation are covered in Section 2. The optimal/suboptimal distributed consensus estimation algorithms are presented in Section 3, respectively. A formal stability analysis procedure is performed in Section 4. A simulation experiment is executed in Section 5, and conclusions are provided in Section 6.

**Notation 1.**  *Throughout the paper, the notations are absolutely standard. $R^{m}$ means the m-dimensional Euclidean space. $A^{T}$ denotes the transpose of the variable A, and $B^{-1}$ represents the inverse matrix of an invertible n×n matrix B. In addition, tr(C) is the trace of the matrix C. E{D} expresses the expectation of the random variable D. diag{•} is a diagonal matrix, and the random variable X has a probability density function denoted by P{X}. N(μ,R) denotes the Gaussian stochastic process with μ and R representing the corresponding mean value and covariance matrix, respectively.*

## 2. Problem Statement

### 2.1. System Description

This paper considers a class of linear time-invariant systems as

(1) $$\begin{array}{cc} x_{k+1}= & Ax_{k}+w_{k} \end{array}$$

(2) $$\begin{array}{cc} \;y_{i,k}= & H_{i}x_{k}+v_{i,k},\;i=1,2,\cdots ,M \end{array}$$

where $x_{k}\in R^{N}$ and $y_{i,k}\in R^{M}$ are the state vector and measurement vector of the *i*-th sensor at time instant *k*, respectively. *A* and $H_{i}$ denote the system and measurement matrices, respectively. In addition, the random variables $w_{k}$ and $v_{i,k}$ are the system noise and the measurement noise respectively, which are assumed to be mutually independent, and satisfy $w_{k}∼N\left(0,Q_{k}\right)$ and $v_{i,k}∼N\left(0,R_{i,k}\right)$.

In practical applications, due to sensor failure, unsuccessful measurement, or network congestion, etc., the measurement values from sensors are not always consecutive and may be randomly lost [27]. Therefore, the missing measurement model in this paper is described as follows:

(3) $$\begin{array}{c} z_{i,k}=\;\gamma_{i,k}H_{i}x_{k}+v_{i,k} \end{array}$$

where the random variable $\gamma_{i,k}$ that satisfies the Bernoulli distribution is used to describe the missing measurement phenomenon, which is assumed to be uncorrelated with all noise signals. Furthermore, the probability density function of $\gamma_{i,k}$ is

$$\begin{array}{cc} P\{\gamma_{i,k}=0\}=\; & 1-\lambda_{i}\\ P\{\gamma_{i,k}=1\}=\; & \lambda_{i} \end{array}$$

where $\lambda_{i}$ represents the probability that the measurement information of the *i*-th sensor successfully arrives.

### 2.2. Hybrid Attack Model

To characterize the communication topology of the above sensor network in Equations (1) and (2), consider a fixed undirected graph $G=(V_{x},E_{x})$ with a set of nodes $V_{x}=\left\{v_{1},v_{2},\cdots ,v_{n}\right\}$ and a set of edges $E_{x}\subseteq V_{x}\times V_{x}$. In this sensor network, the neighbor set of node *i* is defined as $N_{i}=\left\{j|\left(i,j\right)\in E_{x}\right\}$, where the total number of neighbors of node *i*, also called its degree, is expressed as $d_{i}=\left|N_{i}\right|$.

Derived from previous works in [28], a distributed consensus estimation algorithm is introduced for the above sensor network:

(4) $$\begin{array}{c} {\hat{x}}_{i,k+1}=\;A{\hat{x}}_{i,k}+K_{i,k}\left(y_{i,k}-H_{i}{\hat{x}}_{i,k}\right)+\epsilon A\sum_{j\in N_{i}}\left({\hat{x}}_{j,k}-{\hat{x}}_{i,k}\right) \end{array}$$

where ${\hat{x}}_{i,k}$ is the estimate of the state $x_{k}$ for node *i* at time instant *k*. $K_{i,k}$ is the filter gain matrix to be determined, and ϵ is the consensus gain coefficient. Referring to existing works [29,30], it is noted that $\epsilon \in (0,\frac{1}{\delta })$ with $\delta =\max_{i}d_{i}$.

When executing the state estimation process under the distributed consensus estimation in (4), it can be found that not only the innovation of node *i* itself is utilized, but also the estimation information from node *j* needs to be integrated. This prompts us to investigate the security of the estimation information transmitted between nodes *i* and *j* since the information may be subject to various malicious cyber attacks. Therefore, in order to describe the actual cyber attack characteristics more realistically in this paper, the following hybrid attack model is constructed as

(5) $$\begin{array}{c} {\hat{x}}_{j,k}^{a}=\alpha_{ij,k}\left({\hat{x}}_{j,k}+q_{ij,k}b_{ij,k}\right)+\left(1-\alpha_{ij,k}\right)A{\hat{x}}_{j,k-1}^{a} \end{array}$$

where ${\hat{x}}_{j,k}^{a}$ denotes the state estimation for node *j* under hybrid attacks. The random variable $\alpha_{ij,k}$ is used to characterize the occurrence of DoS attacks, which satisfies the Bernoulli distribution. In other words, $\alpha_{ij,k}=0$ means that the estimation information ${\hat{x}}_{j,k}$ is subject to DoS attacks and cannot be successfully transmitted; $\alpha_{ij,k}=1$ indicates otherwise. Furthermore, in the case of DoS attacks, this paper introduces the compensation strategy in [31,32] to improve the loss of transmitted data. $q_{ij,k}$ indicates whether the estimation information transmitted between nodes *i* and *j* is subject to FDI attacks, taking values of 0 or 1. The random variable $b_{ij,k}∼N\left(0,B_{ij,k}\right)$ is used to model FDI attacks, which is also assumed to be uncorrelated with all noise signals.

### 2.3. Problem Statement

In terms of that, this paper considers the issues of missing measurements in (3) and hybrid attacks in (5), so the distributed consensus estimation algorithm in (4) is redesigned as

(6) $$\begin{array}{c} {\hat{x}}_{i,k+1}=\;A{\hat{x}}_{i,k}+K_{i,k}\left(z_{i,k}-\lambda_{i}H_{i}{\hat{x}}_{i,k}\right)+\epsilon A\sum_{j\in N_{i}}\left({\hat{x}}_{j,k}^{a}-{\hat{x}}_{i,k}\right) \end{array}$$

The main goal of this paper, as indicated by the discussion above, is to derive a suitable distributed consensus estimator to estimate the system states under the dual impact of missing measurements and hybrid attacks, and then seek sufficient conditions to ensure the stability of the proposed distributed estimator.

## 3. Distributed Consensus Estimator

In this section, a suitable Kalman filter gain matrix and error covariance matrix are derived to obtain the state estimates.

For the convenience of presentation, first define

$$\begin{array}{cc} e_{i,k} & =\;x_{k}-{\hat{x}}_{i,k},\;e_{j,k}^{a}=\;x_{k}-{\hat{x}}_{j,k}^{a}\\ P_{ij,k} & =\;E\left\{e_{i,k}e_{j,k}^{T}\right\},\;{\hat{P}}_{ij,k}=\;E\left\{e_{i,k}^{a}e_{j,k}^{T}\right\}\\ {\overset{ˇ}{P}}_{ij,k} & =\;E\left\{e_{i,k}{\left(e_{j,k}^{a}\right)}^{T}\right\},\;P_{ij,k}^{a}=\;E\left\{e_{i,k}^{a}{\left(e_{j,k}^{a}\right)}^{T}\right\} \end{array}$$

**Theorem 1.**  *Consider the linear time-invariant system in (1) and (2) under missing measurements in (3) and hybrid attacks in (5). Then, the distributed consensus estimation algorithm designed in (6) has the optimal filter gain as follows:*

$$\begin{array}{cc} K_{i,k}= & \;\lambda_{i}A\left[P_{i,k}+\epsilon \sum_{r\in N_{i}}\left({\hat{P}}_{ri,k}-P_{i,k}\right)\right]H_{i}^{T}G_{i,k}^{-1} \end{array}$$

*where $G_{i,k}=\lambda_{i}^{2}H_{i}P_{i,k}H_{i}^{T}+{\tilde{\lambda }}_{i}H_{i}\Lambda_{k}H_{i}^{T}+R_{i,k}$.*

**Proof of Theorem 1.**  According to the above definition, it can be known

(7) $$\begin{array}{cc} e_{i,k+1}= & \;x_{k+1}-{\hat{x}}_{i,k+1}\\ = & \;\left(A-\lambda_{i}K_{i,k}H_{i}\right)e_{i,k}-K_{i,k}\left(\gamma_{i,k}-\lambda_{i}\right)H_{i}x_{k}\\ \; & \;+\epsilon A\sum_{j\in N_{i}}\left(e_{j,k}^{a}-e_{i,k}\right)+w_{k}-K_{i,k}v_{i,k} \end{array}$$

where

(8) $$\begin{array}{cc} e_{j,k}^{a}= & \;x_{k}-{\hat{x}}_{j,k}^{a}\\ = & \;\alpha_{ij,k}e_{j,k}-\alpha_{ij,k}q_{ij,k}b_{ij,k}+\left(1-\alpha_{ij,k}\right)Ae_{j,k-1}^{a}\\ \; & \;+\left(1-\alpha_{ij,k}\right)w_{k-1} \end{array}$$

Naturally, we can easily obtain the error covariance matrix

(9) $$\begin{array}{cc} P_{ij,k+1}= & \;E\{e_{i,k+1}e_{j,k+1}^{T}\}\\ = & \;\left(A-\lambda_{i}K_{i,k}H_{i}\right)P_{ij,k}{\left(A-\lambda_{j}K_{j,k}H_{j}\right)}^{T}+K_{i,k}H_{i}\\ \; & \;\times E\left\{\left(\gamma_{i,k}-\lambda_{i}\right)\left(\gamma_{j,k}-\lambda_{j}\right)x_{k}x_{k}^{T}\right\}H_{j}^{T}K_{j,k}^{T}+\epsilon^{2}\\ \; & \;\times A\sum_{r\in N_{i}}\sum_{s\in N_{j}}\left(P_{rs,k}^{a}-{\hat{P}}_{rj,k}-{\overset{ˇ}{P}}_{is,k}+P_{ij,k}\right)A^{T}\\ \; & \;+\epsilon \left(A-\lambda_{i}K_{i,k}H_{i}\right)\sum_{s\in N_{j}}\left({\overset{ˇ}{P}}_{is,k}-P_{ij,k}\right)A^{T}\\ \; & \;+\epsilon A\sum_{r\in N_{i}}\left({\hat{P}}_{rj,k}-P_{ij,k}\right){\left(A-\lambda_{j}K_{j,k}H_{j}\right)}^{T}\\ \; & \;+K_{i,k}E\left\{v_{i,k}v_{j,k}^{T}\right\}K_{j,k}^{T}+Q_{k} \end{array}$$

When i=j, it yields

(10) $$\begin{array}{cc} P_{i,k+1}= & \;\left(A-\lambda_{i}K_{i,k}H_{i}\right)P_{i,k}{\left(A-\lambda_{i}K_{i,k}H_{i}\right)}^{T}+{\tilde{\lambda }}_{i}K_{i,k}\\ \; & \;\times H_{i}\Lambda_{k}H_{i}^{T}K_{i,k}^{T}+\epsilon^{2}A\sum_{r\in N_{i}}\sum_{s\in N_{i}}(P_{rs,k}^{a}-{\hat{P}}_{ri,k}\\ \; & \;-{\overset{ˇ}{P}}_{is,k}+P_{i,k})A^{T}+K_{i,k}R_{i,k}K_{i,k}^{T}+Q_{k}\\ \; & \;+\epsilon \left(A-\lambda_{i}K_{i,k}H_{i}\right)\sum_{s\in N_{i}}\left({\overset{ˇ}{P}}_{is,k}-P_{i,k}\right)A^{T}\\ \; & \;+\epsilon A\sum_{r\in N_{i}}\left({\hat{P}}_{ri,k}-P_{i,k}\right){\left(A-\lambda_{i}K_{i,k}H_{i}\right)}^{T} \end{array}$$

where ${\tilde{\lambda }}_{i}=E\left\{\left(\gamma_{i,k}-\lambda_{i}\right)\left(\gamma_{i,k}-\lambda_{i}\right)\right\}=\lambda_{i}\left(1-\lambda_{i}\right)$, and $\Lambda_{k}=E\left\{x_{k}x_{k}^{T}\right\}=A\Lambda_{k-1}A^{T}+Q_{k-1}$.

In addition, it has

(11) $$\begin{array}{cc} {\hat{P}}_{ri,k}= & \;E\{e_{r,k}^{a}e_{i,k}^{T}\}\\ = & \alpha_{i}P_{ri,k}+\left(1-\alpha_{i}\right)[A{\hat{P}}_{ri,k-1}{\left(A-\lambda_{i}K_{i,k-1}H_{i}\right)}^{T}\\ \; & +\epsilon A\sum_{s\in N_{i}}\left(P_{rs,k-1}^{a}-{\hat{P}}_{ri,k-1}\right)A^{T}+Q_{k-1}] \end{array}$$

(12) $$\begin{array}{cc} {\overset{ˇ}{P}}_{is,k}= & \;E\{e_{i,k}{\left(e_{s,k}^{a}\right)}^{T}\}\\ = & \alpha_{i}P_{is,k}+\left(1-\alpha_{i}\right)[\left(A-\lambda_{i}K_{i,k-1}H_{i}\right){\overset{ˇ}{P}}_{is,k-1}A^{T}\\ \; & +\epsilon A\sum_{r\in N_{i}}\left(P_{rs,k-1}^{a}-{\overset{ˇ}{P}}_{is,k-1}\right)A^{T}+Q_{k-1}] \end{array}$$

where $\alpha_{i}=P\left\{\alpha_{ij,k}=1\right\}$.

From the definition of $P_{ij,k}^{a}$, it follows that

(13) $$\begin{array}{cc} P_{rs,k}^{a}= & \;E\{e_{r,k}^{a}{\left(e_{s,k}^{a}\right)}^{T}\}\\ = & \;\alpha_{i}^{2}P_{rs,k}+\alpha_{i}\left({\overset{ˇ}{P}}_{rs,k}-\alpha_{i}P_{rs,k}\right)+\alpha_{i}\left({\hat{P}}_{rs,k}-\alpha_{i}P_{rs,k}\right)\\ \; & \;+{\left(1-\alpha_{i}\right)}^{2}\left(AP_{rs,k-1}^{a}A^{T}+Q_{k-1}\right)+\alpha_{i}q_{ir,k}B_{ir,k} \end{array}$$

Note that the total estimation error for all nodes is expressed as $\sum_{i=1}^{M}E\{∥x_{k}-{\hat{x}}_{i,k}{∥}^{2}\}$, which is equivalent to $\sum_{i=1}^{M}\mathrm{tr}\left(P_{i,k}\right)$. Based on this, the optimal filter gain matrix $K_{i,k}$ can be obtained by solving the equation $\partial \mathrm{tr}\left(P_{i,k+1}\right)/\partial K_{i,k}=0$.

Thus, applying the matrix calculus operation theory yields

(14) $$\begin{array}{cc} \frac{\partial \mathrm{tr}(P_{i,k+1})}{\partial K_{i,k}}= & \;2\left(A-\lambda_{i}K_{i,k}H_{i}\right)P_{i,k}{\left(-\lambda_{i}H_{i}\right)}^{T}\\ \; & +2{\tilde{\lambda }}_{i}K_{i,k}H_{i}\Lambda_{k}H_{i}^{T}+2K_{i,k}R_{i,k}\\ \; & +\epsilon A\sum_{s\in N_{i}}{\left({\overset{ˇ}{P}}_{is,k}-P_{i,k}\right)}^{T}{\left(-\lambda_{i}H_{i}\right)}^{T}\\ \; & +\epsilon A\sum_{r\in N_{i}}\left({\hat{P}}_{ri,k}-P_{i,k}\right){\left(-\lambda_{i}H_{i}\right)}^{T}=0 \end{array}$$

From Equation (14), we have

(15) $$\begin{array}{cc} K_{i,k}= & \;\lambda_{i}A\left[P_{i,k}+\epsilon \sum_{r\in N_{i}}\left({\hat{P}}_{ri,k}-P_{i,k}\right)\right]H_{i}^{T}G_{i,k}^{-1} \end{array}$$

where $G_{i,k}=\lambda_{i}^{2}H_{i}P_{i,k}H_{i}^{T}+{\tilde{\lambda }}_{i}H_{i}\Lambda_{k}H_{i}^{T}+R_{i,k}$. This finishes the proof of Theorem 1. □

## 4. A Scalable Estimation Algorithm and Stability Analysis

Note that the derived error covariance matrix in (10) for the proposed distributed consensus estimation algorithm is not scalable in the number of nodes, making it unsuitable for large-scale systems such as smart grids and mobile communication networks [33]. In order to compensate for this weakness, this paper derives the following suboptimal estimation method:

(16) $$\begin{array}{cc} {\hat{x}}_{i,k+1}= & \;A{\hat{x}}_{i,k}+K_{i,k}\left(z_{i,k}-\lambda_{i}H_{i}{\hat{x}}_{i,k}\right)+\epsilon A\sum_{j\in N_{i}}\left({\hat{x}}_{j,k}^{a}-{\hat{x}}_{i,k}\right)\\ {\hat{x}}_{j,k}^{a}= & \;\alpha_{ij,k}\left({\hat{x}}_{j,k}+q_{ij,k}b_{ij,k}\right)+\left(1-\alpha_{ij,k}\right)A{\hat{x}}_{j,k-1}^{a}\\ K_{i,k}= & \;\lambda_{i}AP_{i,k}H_{i}^{T}{\left(\lambda_{i}^{2}H_{i}P_{i,k}H_{i}^{T}\;+\;{\tilde{\lambda }}_{i}H_{i}\Lambda_{k}H_{i}^{T}+R_{i,k}\right)}^{-1}\\ P_{i,k+1}= & \;\left(A-\lambda_{i}K_{i,k}H_{i}\right)P_{i,k}{\left(A-\lambda_{i}K_{i,k}H_{i}\right)}^{T}+{\tilde{\lambda }}_{i}K_{i,k}\\ \; & \;\times H_{i}\Lambda_{k}H_{i}^{T}K_{i,k}^{T}+K_{i,k}R_{i,k}K_{i,k}^{T}+Q_{k} \end{array}$$

**Remark 1.**  *Such an assumption is achieved by eliminating the influence of the cross-covariance matrices in the proposed distributed consensus estimation algorithm. Instead, by setting ϵ=0 in solving Equations (10) and (15) in this paper, a scalable estimator in (16) is obtained. Meanwhile, it can be easily known that the designed estimator is suboptimal due to the missing terms.*

In the following, a formal stability analysis for the suboptimal distributed consensus estimator constructed as (16) is presented. The following assumptions and lemmas are first given as

**Assumption 1.**  *For some positive numbers, the following inequalities are satisfied*

$$\begin{array}{cc} \underline{f}\leq & ∥A∥\leq \bar{f},\;{\underline{h}}_{i}\leq ∥H_{i}∥\leq {\bar{h}}_{i}\\ \underline{q}I\leq & Q_{k}\leq \bar{q}I,\;{\underline{r}}_{i}I\leq R_{i,k}\leq {\bar{r}}_{i}I\\ \; & {\underline{p}}_{i}I\leq P_{i,k}\leq {\bar{p}}_{i}I \end{array}$$

**Lemma 1**  ([34])**.** *There are real numbers $\bar{\nu },\underline{\nu },\upsilon >0$ and 0<σ≤1 such that the stochastic process $V_{k}\left(\xi_{k}\right)$ satisfies the following inequalities*

(17) $$\begin{array}{c} \underline{\nu }∥\xi_{k}{∥}^{2}\leq V_{k}\left(\xi_{k}\right)\leq \bar{\nu }{∥\xi_{k}∥}^{2} \end{array}$$

*and*

(18) $$\begin{array}{c} E\left\{V_{k+1}\left(\xi_{k+1}\right)|\xi_{k}\right\}-V_{k}\left(\xi_{k}\right)\leq \upsilon -\sigma V_{k}\left(\xi_{k}\right) \end{array}$$

*which means that the stochastic process $V_{k}\left(\xi_{k}\right)$ is exponentially bounded in mean square, and is bounded with probability one.*

Then, the following will present the main results of the stability analysis.

**Theorem 2.**  *For the linear time-invariant system in (1) and (2), consider missing measurements in (3) and hybrid attacks in (5) and the suboptimal distributed consensus estimation algorithm proposed in (16). Under Assumption 1 and setting the following condition*

$$\begin{array}{c} \sum_{j\in N_{i}}{\left({\hat{x}}_{j,k}^{a}-{\hat{x}}_{i,k}\right)}^{T}\left({\hat{x}}_{j,k}^{a}-{\hat{x}}_{i,k}\right)\leq \varsigma_{i} \end{array}$$

*where $\varsigma_{i}>0,i=1,2,\cdots ,n$, the estimation error $e_{i,k}$ is exponentially bounded in mean square and is bounded with probability one.*

**Proof of Theorem 2.**  In order to satisfy the conditions of Lemma 1, first construct the augmented estimation error as $e_{k}=\left[e_{1,k}^{T},e_{2,k}^{T},\cdots ,e_{n,k}^{T}\right]$ and the augmented estimation error covariance as $P_{k}=\mathrm{diag}\left\{P_{1,k},P_{2,k},\cdots ,P_{n,k}\right\}$, and then define a suitable Lyapunov function as follows

(19) $$\begin{array}{c} V_{k}\left(e_{k}\right)=e_{k}^{T}P_{k}^{-1}e_{k}=\sum_{i=1}^{n}e_{i,k}^{T}P_{i,k}^{-1}e_{i,k} \end{array}$$

By Assumption 1, it can be easily obtained

(20) $$\begin{array}{c} \frac{1}{\bar{p}}∥e_{k}{∥}^{2}\leq V_{k}\left(e_{k}\right)\leq \frac{1}{\underline{p}}{∥e_{k}∥}^{2} \end{array}$$

which proves that the first condition (17) of Lemma 1 is satisfied with $\underline{\nu }=\frac{1}{\bar{p}}$ and $\bar{\nu }=\frac{1}{\underline{p}}$. Here, $\bar{p}=\max\left\{{\bar{p}}_{1},{\bar{p}}_{2},\cdots ,{\bar{p}}_{n}\right\}$ and $\underline{p}=\min\left\{{\underline{p}}_{1},{\underline{p}}_{2},\cdots ,{\underline{p}}_{n}\right\}$.

To further meet the second requirement for Lemma 1, Equation (19) needs to be extended. Combining Equation (7), the following expression is obtained:

(21) $$\begin{array}{cc} E\{V_{k+1}\left(e_{k+1}\right)\}=\; & \sum_{i=1}^{n}E\{e_{i,k}^{T}{\left(A-\lambda_{i}K_{i,k}H_{i}\right)}^{T}P_{i,k+1}^{-1}\left(A-\lambda_{i}K_{i,k}H_{i}\right)\\ \; & \;\times e_{i,k}\}+\sum_{i=1}^{n}E\left\{{\tilde{\lambda }}_{i}x_{k}^{T}H_{i}^{T}K_{i,k}^{T}P_{i,k+1}^{-1}K_{i,k}H_{i}x_{k}\right\}\\ \; & \;+\epsilon^{2}\sum_{i=1}^{n}E\{\sum_{j\in N_{i}}{\left(e_{j,k}^{a}-e_{i,k}\right)}^{T}A^{T}P_{i,k+1}^{-1}A\sum_{j\in N_{i}}(e_{j,k}^{a}\\ \; & \;-e_{i,k})\}+2\epsilon \sum_{i=1}^{n}E\{e_{i,k}^{T}{\left(A-\lambda_{i}K_{i,k}H_{i}\right)}^{T}P_{i,k+1}^{-1}\\ \; & \;\times A\sum_{j\in N_{i}}\left(e_{j,k}^{a}-e_{i,k}\right)\}+\sum_{i=1}^{n}E\left\{w_{k}^{T}P_{i,k+1}^{-1}w_{k}\right\}\\ \; & \;+\sum_{i=1}^{n}E\left\{v_{i,k}^{T}K_{i,k}^{T}P_{i,k+1}^{-1}K_{i,k}v_{i,k}\right\} \end{array}$$

According to the definition, ${\tilde{\lambda }}_{i}\geq 0$ is horizontally established. Therefore, it follows from Equation (16) and Assumption 1 that

(22) $$\begin{array}{c} ∥K_{i,k}∥=∥\lambda_{i}AP_{i,k}H_{i}^{T}{\left(\lambda_{i}^{2}H_{i}P_{i,k}H_{i}^{T}+{\tilde{\lambda }}_{i}H_{i}\Lambda_{k}H_{i}^{T}+R_{i,k}\right)}^{-1}∥\leq \frac{\bar{f}{\bar{p}}_{i}{\bar{h}}_{i}}{\lambda_{i}{\underline{h}}_{i}^{2}{\underline{p}}_{i}} \end{array}$$

Similarly, according to (16) and (22), we obtain

(23) $$\begin{array}{cc} P_{i,k+1}\geq & \left(A-\lambda_{i}K_{i,k}H_{i}\right)P_{i,k}{\left(A-\lambda_{i}K_{i,k}H_{i}\right)}^{T}+Q_{k}\\ \geq & \left(A-\lambda_{i}K_{i,k}H_{i}\right)\left[P_{i,k}+\frac{\underline{q}}{(\bar{f}+\frac{\bar{f}{\bar{p}}_{i}{\bar{h}}_{i}^{2}}{{\underline{h}}_{i}^{2}{\underline{p}}_{i}}{)}^{2}}\right]{\left(A-\lambda_{i}K_{i,k}H_{i}\right)}^{T} \end{array}$$

Then, it can be further obtained, as from inequality (23),

$$\begin{array}{c} {\left(A-\lambda_{i}K_{i,k}H_{i}\right)}^{T}P_{i,k+1}^{-1}\left(A-\lambda_{i}K_{i,k}H_{i}\right)\leq {\left[1+\frac{\underline{q}}{{\bar{p}}_{i}(\bar{f}+\frac{\bar{f}{\bar{p}}_{i}{\bar{h}}_{i}^{2}}{{\underline{h}}_{i}^{2}{\underline{p}}_{i}}{)}^{2}}\right]}^{-1}P_{i,k}^{-1} \end{array}$$

Therefore, the first term on the right-hand side of Equation (21) can be scaled as

(24) $$\begin{array}{cc} \; & \sum_{i=1}^{n}E\left\{e_{i,k}^{T}{\left(A-\lambda_{i}K_{i,k}H_{i}\right)}^{T}P_{i,k+1}^{-1}\left(A-\lambda_{i}K_{i,k}H_{i}\right)e_{i,k}\right\}\\ \; & \;\leq [1+\frac{\underline{q}}{{\bar{p}}_{i}(\bar{f}+\frac{\bar{f}{\bar{p}}_{i}{\bar{h}}_{i}^{2}}{{\underline{h}}_{i}^{2}{\underline{p}}_{i}}{)}^{2}}{]}^{-1}E\left\{V_{k}\left(e_{k}\right)\right\} \end{array}$$

In addition, from (16), we have

$$\begin{array}{c} P_{i,k+1}\geq {\tilde{\lambda }}_{i}K_{i,k}H_{i}\Lambda_{k}H_{i}^{T}K_{i,k}^{T} \end{array}$$

Then we have

(25) $$\begin{array}{c} \sum_{i=1}^{n}E\left\{{\tilde{\lambda }}_{i}x_{k}^{T}H_{i}^{T}K_{i,k}^{T}P_{i,k+1}^{-1}K_{i,k}H_{i}x_{k}\right\}\leq \sum_{i=1}^{n}E\left\{x_{k}^{T}\Lambda_{k}^{-1}x_{k}\right\}=n \end{array}$$

Further, we proceed to deal with the other terms in (21). Under Assumption 1, we have

(26) $$\begin{array}{cc} \; & \epsilon^{2}\sum_{i=1}^{n}E\left\{\sum_{j\in N_{i}}{\left(e_{j,k}^{a}-e_{i,k}\right)}^{T}A^{T}P_{i,k+1}^{-1}A\sum_{j\in N_{i}}\left(e_{j,k}^{a}-e_{i,k}\right)\right\}\\ \; & \;\leq \frac{\epsilon^{2}{\bar{f}}^{2}}{{\underline{p}}_{i}}\sum_{i=1}^{n}\sum_{j\in N_{i}}{\left(e_{j,k}^{a}-e_{i,k}\right)}^{T}\left(e_{j,k}^{a}-e_{i,k}\right)\\ \; & \;=\frac{\epsilon^{2}{\bar{f}}^{2}}{{\underline{p}}_{i}}\sum_{i=1}^{n}\sum_{j\in N_{i}}{\left({\hat{x}}_{j,k}^{a}-{\hat{x}}_{i,k}\right)}^{T}\left({\hat{x}}_{j,k}^{a}-{\hat{x}}_{i,k}\right) \end{array}$$

Choose a condition as

(27) $$\begin{array}{c} \sum_{j\in N_{i}}{\left({\hat{x}}_{j,k}^{a}-{\hat{x}}_{i,k}\right)}^{T}\left({\hat{x}}_{j,k}^{a}-{\hat{x}}_{i,k}\right)\leq \varsigma_{i} \end{array}$$

where $\varsigma_{i}>0,i=1,2,\cdots ,n$ is a real number. After that, (26) can be scaled as

(28) $$\begin{array}{c} \epsilon^{2}\sum_{i=1}^{n}E\left\{\sum_{j\in N_{i}}{\left(e_{j,k}^{a}-e_{i,k}\right)}^{T}A^{T}P_{i,k+1}^{-1}A\sum_{j\in N_{i}}\left(e_{j,k}^{a}-e_{i,k}\right)\right\}\leq \frac{\epsilon^{2}{\bar{f}}^{2}}{{\underline{p}}_{i}}\sum_{i=1}^{n}\varsigma_{i} \end{array}$$

In terms of the elementary inequality $x^{T}y+xy^{T}\leq x^{T}x+y^{T}y$, it naturally follows that

(29) $$\begin{array}{cc} \; & 2\epsilon \sum_{i=1}^{n}E\left\{e_{i,k}^{T}{\left(A-\lambda_{i}K_{i,k}H_{i}\right)}^{T}P_{i,k+1}^{-1}A\sum_{j\in N_{i}}\left(e_{j,k}^{a}-e_{i,k}\right)\right\}\\ \; & \;\leq \epsilon \sum_{i=1}^{n}E\left\{e_{i,k}^{T}{\left(A-\lambda_{i}K_{i,k}H_{i}\right)}^{T}P_{i,k+1}^{-1}\left(A-\lambda_{i}K_{i,k}H_{i}\right)e_{i,k}\right\}\\ \; & \;+\epsilon \sum_{i=1}^{n}\sum_{j\in N_{i}}E\left\{{\left(e_{j,k}^{a}-e_{i,k}\right)}^{T}P_{i,k+1}^{-1}\left(e_{j,k}^{a}-e_{i,k}\right)\right\}\\ \; & \;\leq \epsilon {\left[1+\frac{\underline{q}}{{\bar{p}}_{i}(\bar{f}+\frac{\bar{f}{\bar{p}}_{i}{\bar{h}}_{i}^{2}}{{\underline{h}}_{i}^{2}{\underline{p}}_{i}}{)}^{2}}\right]}^{-1}E\left\{V_{k}\left(e_{k}\right)\right\}+\epsilon \sum_{i=1}^{n}\varsigma_{i} \end{array}$$

The remaining noise terms will be processed next, and we have

(30) $$\begin{array}{c} \sum_{i=1}^{n}E\left\{w_{k}^{T}P_{i,k+1}^{-1}w_{k}\right\}\leq \frac{1}{{\underline{p}}_{i}}\sum_{i=1}^{n}E\left\{\mathrm{tr}\left(w_{k}w_{k}^{T}\right)\right\}\leq \frac{\bar{q}Nn}{{\underline{p}}_{i}} \end{array}$$

and

(31) $$\begin{array}{c} \sum_{i=1}^{n}E\left\{v_{i,k}^{T}K_{i,k}^{T}P_{i,k+1}^{-1}K_{i,k}v_{i,k}\right\}\leq \frac{{\bar{f}}^{2}{\bar{p}}_{i}^{2}{\bar{h}}_{i}^{2}}{\lambda_{i}^{2}{\underline{h}}_{i}^{4}{\underline{p}}_{i}^{3}}\sum_{i=1}^{n}E\left\{\mathrm{tr}\left(v_{i,k}v_{i,k}^{T}\right)\right\}\leq \frac{{\bar{f}}^{2}{\bar{p}}_{i}^{2}{\bar{h}}_{i}^{2}{\bar{r}}_{i}Mn}{\lambda_{i}^{2}{\underline{h}}_{i}^{4}{\underline{p}}_{i}^{3}} \end{array}$$

According to Equations (21), (24), (25) and (28)–(31) can be further scaled as

(32) $$\begin{array}{c} E\left\{V_{k+1}\left(e_{k+1}\right)\right\}\leq \upsilon +\left(1-\sigma \right)E\left\{V_{k}\left(e_{k}\right)\right\} \end{array}$$

where

$$\begin{array}{cc} \sigma = & 1-\left(1+\epsilon \right)[1+\frac{\underline{q}}{{\bar{p}}_{i}(\bar{f}+\frac{\bar{f}{\bar{p}}_{i}{\bar{h}}_{i}^{2}}{{\underline{h}}_{i}^{2}{\underline{p}}_{i}}{)}^{2}}{]}^{-1}\\ \epsilon = & \left(\frac{\epsilon^{2}{\bar{f}}^{2}}{{\underline{p}}_{i}}+\epsilon \right)\sum_{i=1}^{n}\varsigma_{i}+n+\frac{\bar{q}Nn}{{\underline{p}}_{i}}+\frac{{\bar{f}}^{2}{\bar{p}}_{i}^{2}{\bar{h}}_{i}^{2}{\bar{r}}_{i}Mn}{\lambda_{i}^{2}{\underline{h}}_{i}^{4}{\underline{p}}_{i}^{3}} \end{array}$$

It can be found that the second condition (18) of Lemma 1 is satisfied when $\epsilon <\underline{q}/{\bar{p}}_{i}(\bar{f}+\frac{\bar{f}{\bar{p}}_{i}{\bar{h}}_{i}^{2}}{{\underline{h}}_{i}^{2}{\underline{p}}_{i}}{)}^{2}$. Finally, it can be concluded that the estimation error is bounded with probability one and exponentially bounded in mean square, which completes the proof of Theorem 2. □

## 5. Simulation Results

In this section, a simulation example of the aircraft tracking problem moving in two-dimensional horizontal plane is presented. The state vector is defined as $x_{k}={\left[\zeta_{k},{\dot{\zeta }}_{k},\eta_{k},{\dot{\eta }}_{k}\right]}^{T}$, which consists of position $\left(\zeta_{k},\eta_{k}\right)$ and velocity $\left({\dot{\zeta }}_{k},{\dot{\eta }}_{k}\right)$. The tracking system considered in this section is as described in (1) and (2), where the relevant parameters are defined as follows:

$$\begin{array}{c} A=\left[\begin{array}{cccc} 1 & T & 0 & 0\\ 0 & 1 & 0 & 0\\ 0 & 0 & 1 & T\\ 0 & 0 & 0 & 1 \end{array}\right],\;T=1 \end{array}$$

$$\begin{array}{c} Q_{k}=0.04\left[\begin{array}{cccc} T^{4}/4 & T^{3}/2 & 0 & 0\\ T^{3}/2 & T^{2} & 0 & 0\\ 0 & 0 & T^{4}/4 & T^{3}/2\\ 0 & 0 & T^{3}/2 & T^{2} \end{array}\right] \end{array}$$

**Figure 1:**
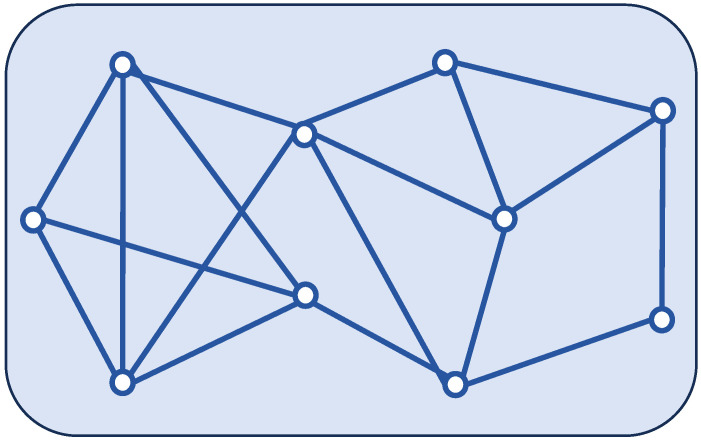
Network topology with M=10.

To track the target aircraft, ten distributed sensors with the topology shown in Figure 1 are utilized, where each sensor interacts with only a matched estimator. The target position is generated as the sensor measurement

$$\begin{array}{cc} y_{i,k}= & \;\left[\begin{array}{cccc} 1 & 0 & 0 & 0\\ 0 & 0 & 1 & 0 \end{array}\right]x_{k}+v_{i,k},\;i=1,2,\cdots ,M \end{array}$$

where the measurement noise covariance is set to $R_{i,k}=i*R_{0}$ with $R_{0}=\mathrm{diag}\left\{0.2^{2},0.2^{2}\right\}$. Further, the initial state is set to $x_{0}={\left[10,1.5,10,1.2\right]}^{T}$.

In addition, the root-mean-square errors (RMSEs) are introduced to more precisely assess the estimation performance. The RMSEs on position and velocity over all sensors are respectively defined as

$$\begin{array}{cc} {\mathrm{RMSE}}_{k}^{p}= & \sqrt{\frac{1}{M}\sum_{t=1}^{M}\left[{\left(\zeta_{k}-{\hat{\zeta }}_{t,k}\right)}^{2}+{\left(\eta_{k}-{\hat{\eta }}_{t,k}\right)}^{2}\right]}\\ {\mathrm{RMSE}}_{k}^{v}= & \sqrt{\frac{1}{M}\sum_{t=1}^{M}\left[{\left({\dot{\zeta }}_{k}-{\hat{\dot{\zeta }}}_{t,k}\right)}^{2}+{\left({\dot{\eta }}_{k}-{\hat{\dot{\eta }}}_{t,k}\right)}^{2}\right]} \end{array}$$

where M=100 represents 100 Monte Carlo runs over 10 targets. ${\hat{\zeta }}_{t,k}$, ${\hat{\eta }}_{t,k}$, ${\hat{\dot{\zeta }}}_{t,k}$, and ${\hat{\dot{\eta }}}_{t,k}$ are the estimates of position and velocity at time *k* from the *i*-th run, respectively.

**Figure 2:**
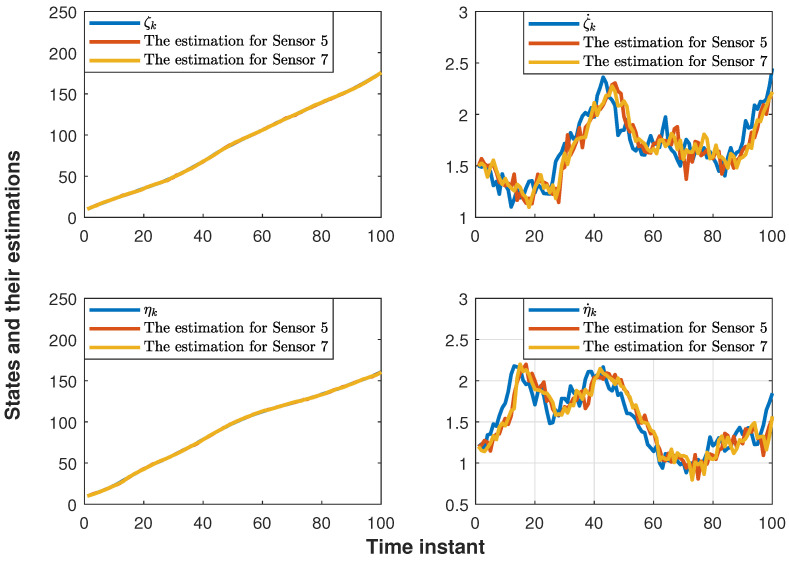
States and their estimations for sensors 5 and 7.

**Figure 3:**
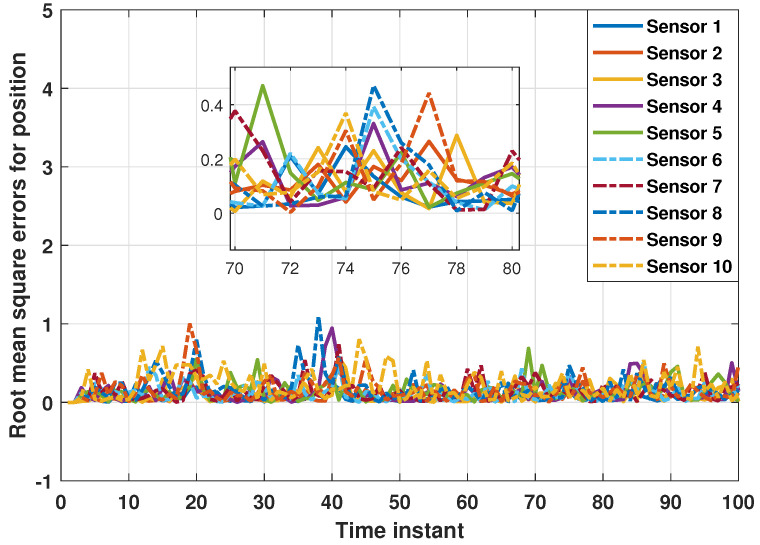
RMSEs in position for ten estimators.

**Figure 4:**
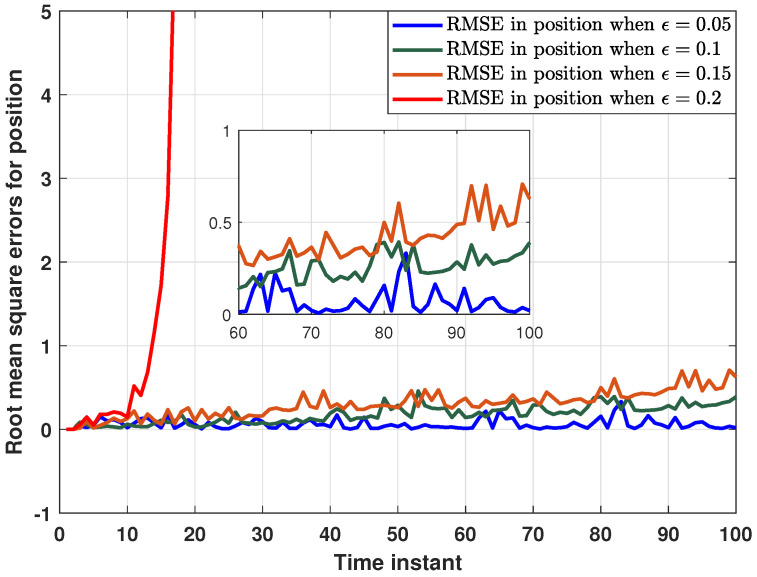
RMSEs in position with different consensus parameter.

The performance of the proposed distributed consensus estimation algorithm under hybrid attacks and missing measurements is shown in Figure 2, where the measurement arrival probability $\lambda_{i}$ is set to 0.9 and the consensus gain is chosen as 0.05. It is assumed that the hybrid attack considered in (5) only occurs between two targets, where the relevant parameters are set to $\alpha_{i}=0.5$ and $B_{ij,k}=0.04I$. It can be found that the distributed consensus estimator still has good tracking performance in position and velocity under hybrid attacks and missing measurements. In addition, the RMSEs in position for ten estimators are presented in Figure 3. It can be seen that all the curves fluctuate around the horizontal axis 0, which also proves the effectiveness of the proposed algorithm in (16). Further, RMSEs in position with different consensus gains ϵ=0.05,0.1,0.15,0.2 are plotted in Figure 4. It can be seen that when ϵ=0.05,0.1,0.15, the curves still fluctuate near the horizontal axis 0, but when ϵ=0.2, the curve rises rapidly. Thus, it can be found that the consensus parameter in this paper has an upper bound. Once this upper bound is exceeded, the estimator lacks stability, which also proves the correctness of Theorem 2. It is worth noting that the consensus parameters need to be chosen very carefully to make a tradeoff between tracking performance and stability.

**Figure 5:**
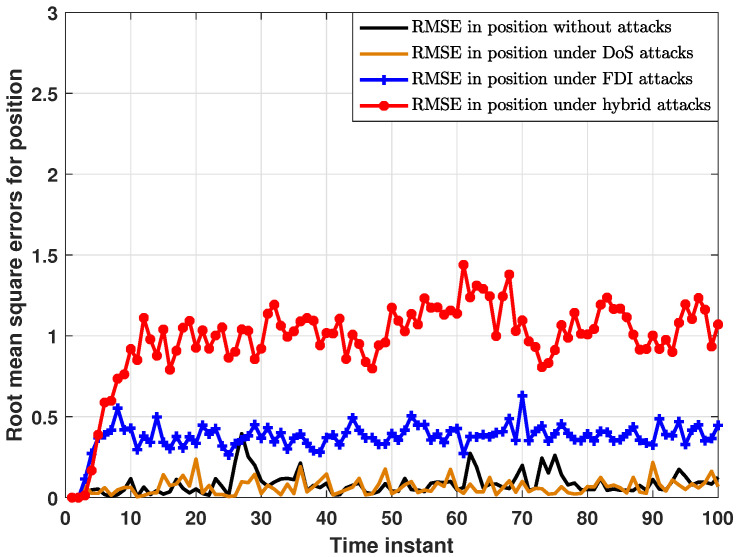
RMSEs in position under hybrid attacks, FDI attacks and DoS attacks as well without attacks.

**Figure 6:**
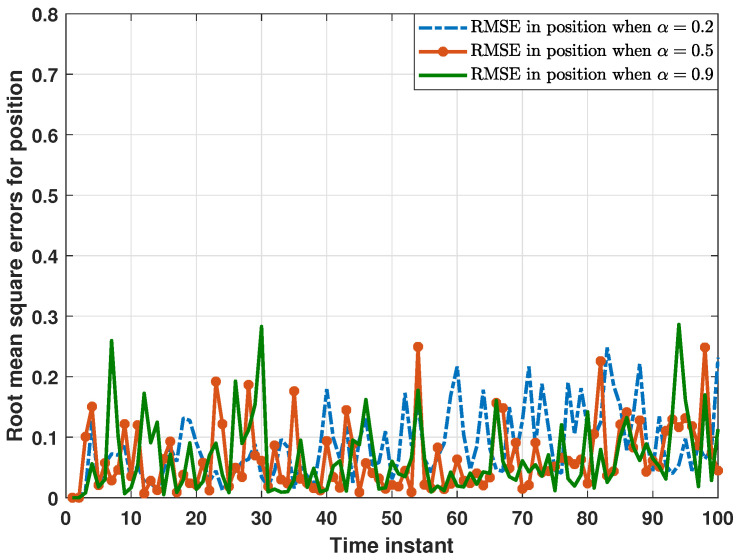
RMSEs in position under different DoS attack probabilities.

**Figure 7:**
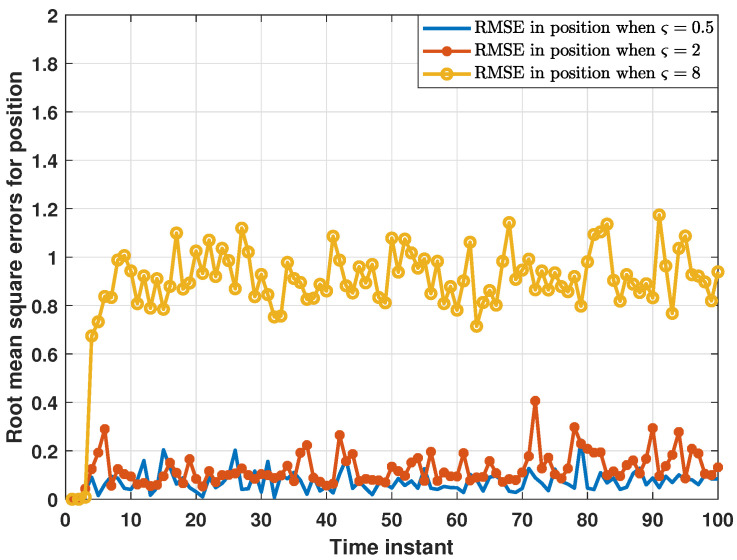
RMSEs in position under different FDI attack intensities.

On the other hand, the performance of the proposed distributed consensus estimation algorithm under hybrid attacks, FDI attacks in [30] and DoS attacks, as well as without attacks in [35] are compared in Figure 5. For examining the impact of different attack scenarios on the proposed distributed estimation algorithm, it is first necessary to exclude the interference of the missing measurement, so the measurement arrival probability $\lambda_{i}$ is set to 1 in this comparison experiment. As shown in Figure 5, RMSEs under hybrid attacks are significantly higher than those under other attack scenarios, while RMSEs under only DoS attacks are almost the same as those without attacks. This proves that hybrid attacks have a greater impact on the proposed distributed estimator than only one single type of attack. In addition, this paper introduces a compensation strategy for DoS attacks. Thus, only DoS attacks have little impact on the estimation performance of the proposed distributed estimator, which is clearly demonstrated in Figure 6. As shown in Figure 6, RMSEs in position do not change significantly as the DoS attack probability increases, strongly proving the effectiveness of the compensation strategy. Further, RMSEs in position under different FDI attack intensities are plotted in Figure 7. In order to better characterize the FDI attack intensity, the standard deviation ς of the random attack variable $b_{ij,k}$ is introduced. It is clear that the proposed distributed consensus estimator has certain resistance to FDI attacks when ς is small. Finally, it is noted that hybrid attacks will degrade the estimation performance in Figure 5, but the distributed estimator proposed in this paper can still remain stable, which is a major advantage of this algorithm.

## 6. Conclusions

In this paper, a modified distributed consensus estimation algorithm has been provided for networked multi-sensor systems subject to hybrid attacks and missing measurements. A random variable satisfying the Bernoulli distribution has been applied to account for the missing measurement phenomenon. From the viewpoint of the attacker, a unified hybrid attack model has been constructed to disrupt the data transmission between neighboring estimators, which takes into account the characteristics and behaviors of both random FDI and DoS attacks. Starting from optimality and scalability, optimal/suboptimal distributed consensus estimators have been proposed, respectively. Furthermore, sufficient conditions for convergence of the proposed distributed suboptimal estimator have been obtained. It has been explicitly established that there are correlations between the convergence and hybrid attack model as well as missing measurement parameters. Future works will focus on extending linear multi-sensor systems to nonlinear systems.

## Data Availability

Data are contained within the article.

## Abbreviations

The following abbreviations are used in this manuscript:

FDI	false data injection
DoS	denial of service
RMSEs	root-mean-square errors
